# Comprehensive proteome, phosphoproteome and kinome characterization of luminal A breast cancer

**DOI:** 10.3389/fonc.2023.1127446

**Published:** 2023-03-31

**Authors:** Ganglong Yang, Chenyang Zuo, Yuxiang Lin, Xiaoman Zhou, Piaopiao Wen, Chairui Zhang, Han Xiao, Meichen Jiang, Morihisa Fujita, Xiao-Dong Gao, Fangmeng Fu

**Affiliations:** ^1^ The Key Laboratory of Carbohydrate Chemistry & Biotechnology, Ministry of Education, School of Biotechnology, Jiangnan University, Wuxi, China; ^2^ State Key Laboratory of Biochemical Engineering, Institute of Process Engineering, Chinese Academy of Sciences, Beijing, China; ^3^ Department of Breast Surgery, Fujian Medical University Union Hospital, Fuzhou, Fujian, China; ^4^ Department of General Surgery, Fujian Medical University Union Hospital, Fuzhou, Fujian, China; ^5^ Breast Cancer Institute, Fujian Medical University, Fuzhou, Fujian, China; ^6^ Department of Pathology, Fujian Medical University Union Hospital, Fuzhou, Fujian, China

**Keywords:** invasive ductal carcinoma (IDC), invasive lobular carcinoma (ILC), proteome, phosphoproteome, kinome, luminal A breast cancer

## Abstract

**Background:**

Breast cancer is one of the most frequently occurring malignant cancers worldwide. Invasive ductal carcinoma (IDC) and invasive lobular carcinoma (ILC) are the two most common histological subtypes of breast cancer. In this study, we aimed to deeply explore molecular characteristics and the relationship between IDC and ILC subtypes in luminal A subgroup of breast cancer using comprehensive proteomics and phosphoproteomics analysis.

**Methods:**

Cancer tissues and noncancerous adjacent tissues (NATs) with the luminal A subtype (ER- and PR-positive, HER2-negative) were obtained from paired IDC and ILC patients respectively. Label-free quantitative proteomics and phosphoproteomics methods were used to detect differential proteins and the phosphorylation status between 10 paired breast cancer and NATs. Then, the difference in protein expression and its phosphorylation between IDC and ILC subtypes were explored. Meanwhile, the activation of kinases and their substrates was also revealed by Kinase-Substrate Enrichment Analysis (KSEA).

**Results:**

In the luminal A breast cancer, a total of 5,044 high-confidence proteins and 3,808 phosphoproteins were identified from 10 paired tissues. The protein phosphorylation level in ILC tissues was higher than that in IDC tissues. Histone H1.10 was significantly increased in IDC but decreased in ILC, Conversely, complement C4-B and Crk-like protein were significantly decreased in IDC but increased in ILC. Moreover, the increased protein expression of Septin-2, Septin-9, Heterogeneous nuclear ribonucleoprotein A1 and Kinectin but reduce of their phosphorylation could clearly distinguish IDC from ILC. In addition, IDC was primarily related to energy metabolism and MAPK pathway, while ILC was more closely involved in the AMPK and p53/p21 pathways. Furthermore, the kinomes in IDC were primarily significantly activated in the CMGC groups.

**Conclusions:**

Our research provides insights into the molecular characterization of IDC and ILC and contributes to discovering novel targets for further drug development and targeted treatment.

## Introduction

Breast cancer is prevalent in women, with approximately 1.7 million cases diagnosed worldwide every year, representing a major leading cause of cancer-related death in women (1). Breast cancer is a heterogeneous disease with diverse clinical behaviors and histopathological patterns, and the heterogeneity of breast cancer biology represents major challenges to the drive for personalized treatment (2). Invasive ductal carcinoma (IDC) and invasive lobular carcinoma (ILC) constitute the two most common histological subtypes of breast cancer, ranging from 66-71% and 6-10% of cases, respectively (3, 4). IDC has played a dominant role in breast cancer, but the incidence of ILC is increasing (5, 6), and studies have evaluated the different clinical characteristics of these subtypes (7, 8). Compared to IDC patients, ILCs present with larger tumor sizes, more frequent lymph node invasion and higher probabilities of estrogen receptor (ER) and progesterone receptor (PR) positivity (9, 10). Clinicopathological analyses indicated that the overall outcome of ILC did not significantly differ from that of the common IDC type (11), and these tumors are currently treated similarly (12, 13). Moreover, studies suggested that the prognosis, long-term outcomes, aggressive and metastasis are different between IDC and ILC even with the specific molecular subgroup (14).

Recently, comprehensive genomic and transcriptomic studies of IDC and ILC have revealed differences between these two subtypes (15–17). Due to alternative splicing, RNA transcripts and proteins are not typically ‘one-to-one’ corresponding relationships (18). Additionally, a variety of protein posttranslational modifications (glycosylation, methylation, phosphorylation, ubiquitination) also contribute to proteoforms exhibiting more diversity than genes (19). Protein phosphorylation is one of the most important posttranslational modifications and plays a key role in protein activity regulation, signal transduction, and intercellular communication (20). Dysregulated protein phosphorylation usually leads to the onset of various malignant diseases, which could be developed as potential candidates for their detection, especially in cancers (21). Protein expression and modification, which directly indicates the pathological status of cancer tissues, have not been evaluated in IDC and ILC. MS-based proteomics has emerged as the most important and powerful tool for protein and phosphorylation analysis. Phosphoproteomics provides additional information, offering a method to qualify and quantify the state of kinase-dependent pathways and provides detailed posttranslational phosphorylation information (22, 23). The activity of kinases, involving the transfer of a phosphate group to a protein, and phosphatases, which remove a phosphate group from a protein, modulate these two enzymatic processes and modulate protein phosphorylation in cells in response to an external stimuli (24). To date, approximately 538 known kinases have been identified in the human genome, most of which are associated with human cancer initiation and progression (21). These kinases maintain cellular function by activating protein function, while corresponding phosphatases reverse this action (25, 26). These counter mechanisms greatly improve the plasticity of the epigenome by regulating protein activity.

In this study, we conducted a comprehensive proteomics and phosphoproteomics analysis on 10 paired luminal A breast cancer patients (5 paired IDC vs 5 paired ILC) to distinguish IDC and ILC from their protein expression levels, their degree of protein phosphorylation and kinase activity. These findings will help clarify the molecular patterns and different pathways of different luminal A breast cancer subtypes and help improve the sensitivity and accuracy of detecting these two subtypes.

## Materials and methods

### Patients and clinical specimens

The paired luminal A breast cancer samples used in this study were obtained from patients who underwent breast surgery (total mastectomy or breast-conserving surgery) in the Department of Breast Surgery, Fujian Medical University Union Hospital. Patients were randomly selected from October 2017 to October 2019. None of the patients underwent any neoadjuvant chemotherapy or radiotherapy prior to surgery. Surgically resected primary tumor tissues and paired noncancerous adjacent tissues (> 3 cm apart from the tumor edge) were collected from 5 patients with invasive ductal carcinoma and 5 patients with invasive lobular carcinoma. All patients were histopathologically diagnosed with the luminal subtype (ER- and PR-positive, HER2-negative; HER2 status was strictly defined according to ASCO guidelines). Clinical information was collected, including age, tumor size, status of axillary lymph node metastasis, AJCC stage and histological grade. Each specimen was collected intraoperatively and immediately transferred to a sterile tube, snap frozen in liquid nitrogen and stored at -80°C until use.

### Tissue homogenization and protein extraction

Frozen tissue samples were homogenized in lysis buffer containing a protease inhibitor cocktail. The lysis buffer contained 8 M urea, 75 mM NaCl, 1 M NH_4_HCO_3_, 50 mM Tris (pH 8.0), 1 mM EDTA, 2 μg/mL aprotinin, 10 μg/mL leupeptin, 1 mM PMSF (Phenylmethanesulfonyl fluoride), 1 mM PIC2, and 1 mM PIC3. All reagents were purchased from Sigma–Aldrich. Phosphatase inhibitor cocktail 2/3 was used at a 1:100 dilution (v/v) with 20 μM PUGNAc (O-(2-Acetamido-2-deoxy-D-glucopyranosylidenamino) N-phenylcarbamate), and 10 mM NaF (sodium fluoride). The tissues were homogenized for 10 min in 25% rated power with 20 s intervals per 10 s sonication using ultrasonic crushing (Scientz, Ningbo, China). The protein concentrations were measured by a bicinchoninic acid (BCA) assay kit (Beyotime, Shanghai, China), and the extracted proteins were stored at -80°C until use.

### Protein digestion by trypsin

Protein (1 mg) was reduced in 10 mM dithiothreitol (DTT, Sigma–Aldrich, U.S.) at 56°C for 45 min and alkylated by the addition of 20 mM iodoacetamide (IAM, Sigma–Aldrich, U.S.) at room temperature for 45 min in the dark. The samples were diluted 8-fold in 40 mM NH_4_HCO_3_. Trypsin (Promega, U.S.) with an enzyme/protein ratio of 1:50 (w/w) was added to the solution and incubated at 37°C overnight. All digested samples were desalted by running through C18 columns (Sep-Pak tC18 cartridge, Waters, U.S.) and dried by Speed-Vac (EYELA-UT-1000, Japan). In the experiment, we routinely obtained over 800 μg of tryptic peptide through digestion and C18 clean-up processes. From the total tryptic peptide, 10% was set apart for conventional global proteomics analysis, while the remaining sample was used for sequential IMAC enrichment. Finally, peptide (1 μg) from each group was subjected to LC−MS/MS on an Orbitrap Fusion Lumos mass spectrometer (Thermo Fisher, U.S.) (27).

### Phosphopeptide enrichment

IMAC enrichment was performed using freshly prepared Fe^3+^-NTA agarose beads that were made by conjugating Ni^2+^-NTA agarose beads (Qiagen, Germany) and FeCl_3_ (Sigma–Aldrich, U.S.) aqueous solution. The Fe^3+^-NTA agarose beads were packaged to the top of C18 stagetip. Each sample was reconstituted in 80% (v/v) acetonitrile (ACN) and 0.1% (v/v) trifluoroacetic acid (TFA) binding/washing buffers and incubated with 25% (v/v) Fe^3+^-NTA agarose beads for 30 min at room temperature. the supernatants were collected by centrifugation. The beads were stacked on the stage tips and washed three times. The phosphopeptides were eluted in potassium phosphate buffer (500 mM KH_2_PO_4_, pH 7) and 50% (v/v) ACN and 0.1% (v/v) formic acid (FA). Then, the peptides were eluted, dried, and stored at -80°C prior to LC–MS/MS analysis of phosphoproteomics (28).

### LC–MS/MS analysis

The dried global peptides and phosphopeptides were resuspended in 10 μL 2% ACN and 0.1% FA solution and then analyzed using an EASY-nLC 1200 system (Thermo Scientific, San Jose, CA) coupled with a high-resolution Orbitrap Fusion Lumos spectrometer (Thermo Scientific, San Jose, CA). Each injection volume was 3 μL. The samples were first separated on an EASY-nLC 1200 system (Thermo Scientific, San Jose, CA) in an RSLC C18 column (1.9 μm×100 μm×20 cm) packed in house. All samples were subjected to two LC−MS/MS runs on an Orbitrap Fusion Lumos mass spectrometer (Thermo Scientific, San Jose, CA). Data-dependent higher-energy collisional dissociation (HCD) fragmentation was performed on the top 20 most abundant ions. For the global peptides, the mobile phase consisting of water (A) and 0.1% FA and 90% ACN (B) was subjected to a gradient elution of 3-8% B, 8 min; 8–28% B, 80 min, 28–32% B, 22 min; 32–80% B, 5 min; and 80% B, 5 min. The flow rate was maintained at 450 nL/min. The spray voltage was in static mode. Spectra (AGC target of 4 × 10 (5) and maximum injection time of 50 ms) were collected from 350 to 2000 m/z at a resolution of 60,000, followed by data-dependent HCD MS/MS (at a resolution of 30,000, HCD collision energy 34%, stepped collision energy 5%, AGC target 5 × 10 (4), maximum injection time 35 ms and microscans 1). Charge state screening was enabled to reject singly charged ions and ions with more than eight charges. A dynamic exclusion time of 45 s was used to discriminate newly selected from previously selected ions. For the phosphopeptides, the gradient elution was 2-22% B, 88 min; 22-32% B, 22 min, 32-80% B, 5 min; and 80% B, 5 min. The flow rate was maintained at 550 nL/min. Spectra (AGC target 4 × 10 (5) and maximum injection time 50 ms) were collected from 350 to 1550 m/z at a resolution of 60,000, followed by data-dependent HCD MS/MS (at a resolution of 30,000, HCD collision energy 32%, stepped collision energy 5%, AGC target 5 × 10 (4), maximum injection time 35 ms and microscans 1).

### Identification of global proteins and phosphoproteins

For the global peptides and phosphopeptides, the acquired MS/MS spectra were searched using MaxQuant software with the Homo sapiens_UniProt database updated in June 2019, which contains 20,432 proteins (29). The parameters were set as default if not otherwise stated. The enzyme specificity was set to trypsin/P (the C-terminus of Arg or Lys with cleavage at the proline bond allowed), and the maximum number of missed cleavage sites was set as two. Carbamidomethyl (C, 43.028) was set as the fixed modification. The deamidation (N, 29.018) and phosphorylation (S/T/Y, 97.977) of proteins were set as the variable modifications. Proteins and peptides were identified using a target-decoy approach in revert mode and quantified using intensity data (peak area of extracted ion chromatography) using the Andromeda search engine integrated into the MaxQuant environment. MaxQuant was searched with a fragment ion mass tolerance of 0.02 Da and a parent ion tolerance of 10.0 PPM. The false discovery rates (FDRs) of protein groups, peptides, and phosphosites were less than 0.01. The cutoff criterion for the localization probability at each phosphosite was greater than 0.75.

### Data processing and visualization

To classify the data, a PCA score plot and correlation heatmap were generated using OmicStudio (https://www.omicstudio.cn/tool). Basic statistical analysis of the data was performed using Excel to obtain information such as the types of identified proteins and phosphoproteins and the number of modified sites. Quantitative analysis of data (standardized analysis and differential protein screening) was performed according to the modified global normalization formula (30). Screening of differential proteins was performed according to the following conditions: P-value < 0.05, fold change >1.50 or <0.67, and significant count pair percentage >60% (more than 3 pairs of the 5 pairs reached a fold change of 1.50 with an accordant trend). Missing values were not included in this quantitative analysis. A two-tailed Welch t-test was conducted to determine the statistical significance of the differences in protein expression or phosphorylation modification between noncancerous and cancer tissues. Volcano plots of differential proteins and phosphoproteins (P-value < 0.05, fold change >1.5 or <0.67) were generated using OmicStudio and R studio (R version 4.0.1, https://www.r-project.org/). Clustering heatmaps were generated using TBtools (https://github.com/CJ-Chen/TBtools) (31). Gene set enrichment analysis (GSEA) was performed to reveal the protein function and involved pathways of the differential proteins using ClusterProfiler (32) and Enrichplot packages (Bioconductor). Gene Ontology (GO) enrichment analysis and KEGG enrichment analysis were performed using R studio, Clusterprofiler packages and Database for Annotation, Visualization and Integrated Discovery (DAVID) (Version 6.8, https://david.ncifcrf.gov/) (33). Significantly enriched pathways were retrieved by searching against KEGG databases. Kinase-substrate enrichment analysis (KSEA) was performed to reveal the activation/inactivation of kinase using the KSEA algorithm (34, 35) using the PhosphoSitePlus database [https://www.phosphosite.org/] according to the following conditions: P-value cutoff < 0.05, substrate count cutoff ≥ 3. An activated-inactivated kinase location analysis plot was generated using the kinMAP app (http://www.kinhub.org/kinmap/citation.html) (36).

## Results

### Protein expression and phosphorylation between IDC and ILC breast cancer

To elucidate differences in the proteome and phosphoproteome profiles of IDC and ILC, we assembled 10 paired breast cancer clinical tissues and classified them into two groups based on their predominant subtype as described in the Materials and Methods section. Patient characteristics, including age, histological type, tumor stage and molecular subtypes, are summarized in **Table 1**.

**Table 1:** Summary of clinical information on the ten subjects involved in this study.

Patient No.	Age	Histological type*	Tumor stage	Lymph node status	Molecular subtype	Luminal subtype^#^		
						ER	PR	HER2
P1	45	IDC	T1	N0	Luminal A	+	+	–
P2	47	IDC	T2	N0	Luminal A	+	+	–
P3	44	IDC	T2	N0	Luminal A	+	+	–
P4	61	IDC	T1	N0	Luminal A	+	+	–
P5	58	IDC	T1	N1	Luminal A	+	+	–
P6	46	ILC	T2	N3	Luminal A	+	+	–
P7	40	ILC	T2	N1	Luminal A	+	+	–
P8	50	ILC	T2	N0	Luminal A	+	+	–
P9	44	ILC	T3	N1	Luminal A	+	+	–
P10	49	ILC	T2	N1	Luminal A	+	+	–

*IDC, invasive ductal carcinoma; ILC, invasive lobular carcinoma;

^#^ER, estrogen receptor; PR, progesterone receptor; HER2, Human epidermal growth factor receptor-2. +, positive; -, negative.

Proteomics and phosphoproteomics data could provide an excellent chance to explore the relationships between protein expression and phosphorylation modification in different cancers. In the proteomics study, a total of 5,044 high-confidence proteins were identified from tumor and noncancerous adjacent tissues (NATs) of 10 paired breast cancer patients, in which the average identified protein number of NATs was lower than that of the cancer tissues (**Figures S1A, B**). Alterations in protein phosphorylation in breast cancer cells and tissues has revealed the heterogeneity of breast cancer (37, 38). However, differences in protein phosphorylation between IDC and ILC are still not very clear. In this study, a total of 3,808 phosphoproteins with 12,552 phosphopeptides were identified from breast cancer tissues. This result indicated that approximately 63% of phosphoproteins were identified in both cancer and NATs, similar to the proteomics results (**Figure S1C**). With respect to protein phosphorylation of the breast cancer subtypes, protein phosphorylation levels in ILC were higher than that of IDC, in which the number of phosphorylation sites in ILC were twofold the number in IDC (**Figure S1D**). The number of peptides with more than one phosphorylation site in IDC cancer tissues was almost equal to the number in NATs, while the number in ILC cancer tissues was markedly higher than that in NATs (**Figure S1E**).

To estimate the functional enrichment of the breast cancer tissue, proteins identified only in the cancer tissues of breast cancer were subjected to GO and KEGG analysis. The results indicated that these proteins were primarily involved in mitochondrial biogenesis gene expression, such as mitochondrial translation elongation and termination (**Figure S2A**). Mitochondria influence multiple processes that underpin tumor progression, including the proliferation of transformed cells, resistance to adverse microenvironmental conditions (39). Moreover, the proteins only in IDC tissues were mainly related with protein-containing complex disassembly while only in ILC tissues were mainly related with cellular component disassembly (**Figures S2B, C**). The KEGG analysis revealed that the proteins found only in cancer tissue were primarily involved the pathways of nucleocytoplasmic transport, endocytosis, and salmonella infection (**Figure S3**). In the IDC, proteins with multiple modified phosphorylation sites were primarily related to the spliceosome and other functions and were enriched only in the insulin signaling and mTOR signaling pathways. In the ILC, proteins with multiple phosphorylation sites were mainly related to various phosphorylation-related signaling pathways, such as the insulin signaling pathway, thyroid hormone signaling pathway, and AMPK signaling pathway (**Figure S4**).

### Protein and phosphorylation features between IDC and ILC breast cancer subtypes

**Figure 1:**
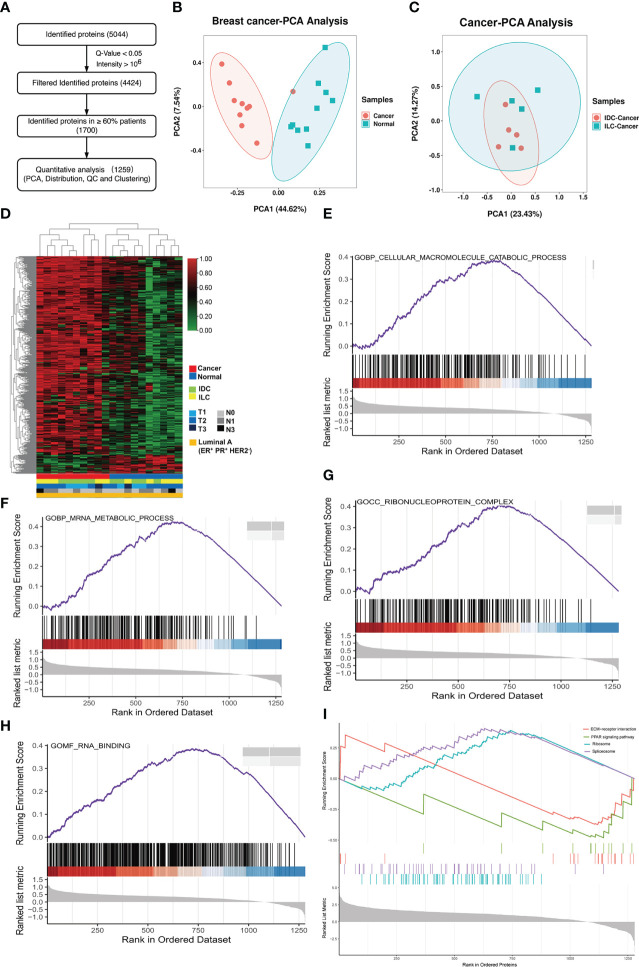
Proteome characterization of luminal A breast cancer. **(A)** Flowchart of data filter and normalization analysis of proteins identified from luminal A breast cancer. **(B)** PCA of 1,259 proteins in 10 paired luminal A breast cancer tissues. Red: tumor, and green: NAT. **(C)** PCA of 1,259 proteins identified from the cancer and NATs tissues. Red: IDC cancer, and green: ILC cancer. **(D)** Heatmap of proteins in tumors and NATs and their associated clinical information showing expression of proteins in cancer or NAT samples. **(E-H)** GSEA results of luminal A breast cancer tissues revealed Gene Ontology (biological process, cellular component, molecular function) results associated with important metabolic functions. **(I)** GSEA plots revealed KEGG pathways associated with classical cancer functions.

To quantitatively distinguish protein expression between IDC and ILC breast cancer subtypes, 1,259 proteins were identified as high-patient-coverage proteins presented in more than 60% of 10 paired luminal A breast cancer patients (**Figure 1A**). From the relative abundance ratio distribution (fold change of protein relative abundance between the breast cancer tissue and NATs), protein expression was globally upregulated in all cancer tissues compared to NATs, in which the ratio distribution moved to 1 (**Figure S5A**). Correlation analysis of the cancer and NATs proteomics revealed that the Pearson’s correlation parameters of the cancer tissue with all other cancer samples were significantly higher than those of paired NATs (**Figure S5B**). The PCA results showed that the cancer tissues and the NATs of luminal A breast cancer can be clearly classified, but it is difficult to globally distinguish the IDC and ILC subtype of luminal A breast cancer (**Figures 1B, C** and **Figure S6**). The heatmap of the proteins showed that cancer and NAT samples were clustered into two groups, but it was also difficult to distinguish the two subtypes (**Figure 1D**). All these proteins from luminal A breast cancer were enriched in protein translation and transfer biological processes and the cellular macromolecule catabolic process and mRNA metabolic process were upregulated (**Figures 1E, F**). While the ribonucleoprotein complex related proteins and RNA binding related proteins were also upregulated in the cancer tissues (**Figures 1G, H**). The KEGG analysis of proteins indicated relative upregulation of molecular metabolic and biosynthetic process proteins, as well as ribosome and spliceosome pathway proteins in the luminal A breast cancer tissues, which were associated with a decrease in cell-ECM receptor interaction and PPAR signaling pathway proteins (**Figure 1I**).

**Figure 2:**
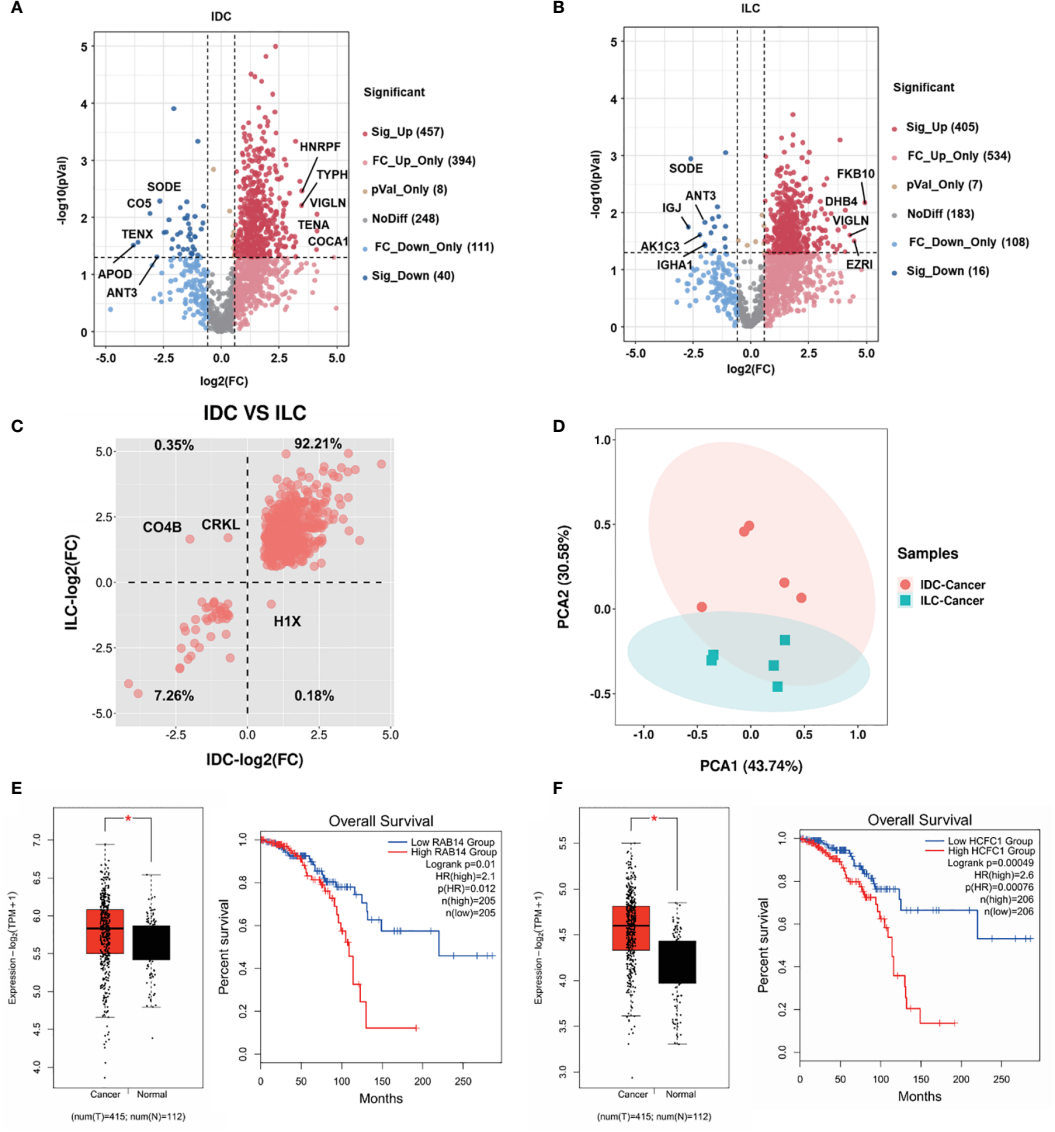
Proteome relative abundance difference and clinical significance of IDC and ILC from luminal A breast cancer. **(A)**, **(B)** Differential proteins between tumors and NATs from the IDC and ILC subtypes. Red: upregulated proteins blue: downregulated proteins (P-value < 0.05 from t-test and fold change > 1.50). **(C)** Intertumor comparative analysis of differential proteins from IDC and ILC. The second quadrant shows that the proteins are downregulated in IDC and upregulated in ILC, and the fourth quadrant shows the opposite. **(D)** PCA of selected proteins in 10 paired cancer tissues. Red: IDC cancer, and green: ILC cancer. **(E-F)** The mRNA expression and Kaplan-Meier survival analysis of significantly differential proteins (Ras-related protein Rab-14: RAB14; Host cell factor 1: HCFC1) in the Luminal A breast cancer using TCGA database. *, p<0.05.

As shown in the volcano plot for different subtypes of breast cancer, 457 proteins in IDC and 405 proteins in ILC were significantly upregulated (fold change, FC>1.5) compared with their NATs. Moreover, 16 proteins in IDC and 40 proteins in ILC were significantly downregulated in ILC (FC<0.67) compared with their NATs. Among the top 5 differential proteins, VIGLN (Vigilin) was upregulated and SODE (extracellular superoxide dismutase) was downregulated in both the IDC and ILC subtypes (**Figures 2A, B**). Furthermore, the differential proteins in IDC and ILC were separated into four groups using the average FC to distinguish the two subtypes. Approximately 99.47% of differential proteins were increased or decreased in both subtypes. Only one protein (H1X, Histone H1.10) was markedly increased in IDC but decreased in ILC, while two proteins (CO4B [Complement C4-B] and CRKL [Crk-like protein]) were obviously decreased in IDC but increased in ILC (**Figure 2C**). Additionally, immunohistochemistry data from the Human Protein Atlas (HPA) were applied to validate expression differences between IDC and ILC (40). Compared to ILC tumor tissues in immunohistochemistry, expression of H1X in IDC was remarkably upregulated, the expression of CRKL was significantly downregulated (http://www.proteinatlas.org/). The verification results basically consistent with our proteomic results. Moreover, these three proteins may represent potential biomarkers to distinguish IDC and ILC from the PCA results (**Figure 2D**). The significantly upregulated proteins RAB14 (Ras-related protein Rab-14) and HCFC1 (Host cell factor 1) in IDC and ILC with the ER- and PR-positive, HER2-negative patients (cancer No. 415 and NATs No. 112) were analyzed from mRNA level based on the TCGA database. It showed that the tendency of mRNA level is similar with the protein expression between the cancer and NATs. From the survival analysis for ER- and PR-positive, HER2-negative patients using TCGA database, patients with RAB14^high^ and HCFC1^high^ had significantly shorter overall survival time than those with RAB14^low^ and HCFC1^low^ (**Figures 2E, F**).

**Figure 3:**
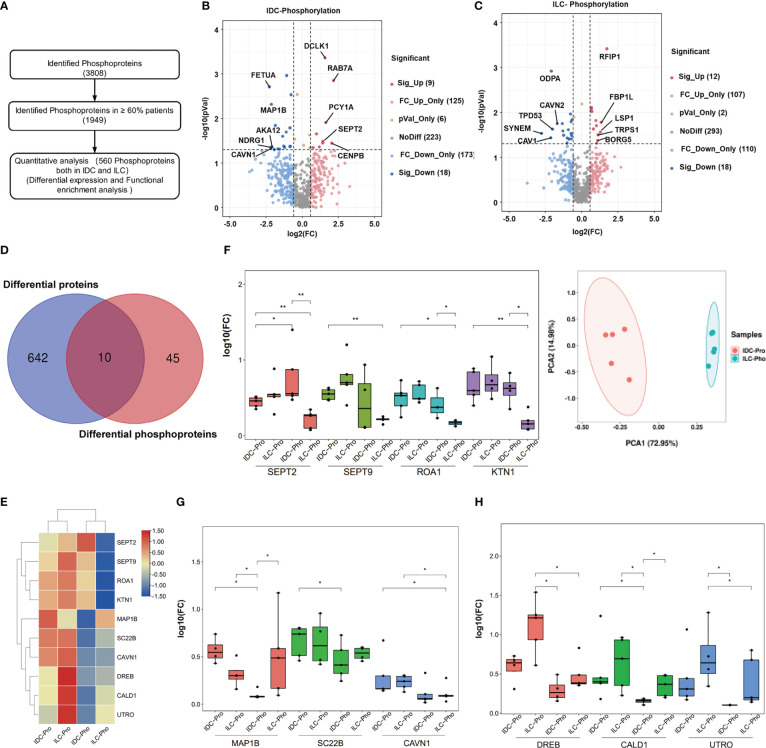
Phosphoproteome characterization of luminal A breast cancer. **(A)** Flowchart of data filter and normalization analysis of phosphoproteins identified from luminal A breast cancer. **(B, C)** Differential phosphoproteins between luminal A breast cancer tissues and their NATs. Red: Upregulated proteins, blue: Downregulated (P-value < 0.05 from t.-test and fold change > 1.50). **(D)** Overlap of differential proteins (Blue) and differential phosphoproteins (Red). **(E)** Protein expression levels and phosphorylation status of 10 selected phosphoproteins in IDC and ILC. **(F)** Left: Expression of SEPT2, SEPT9, ROA1 and KTN1 proteins and their phosphorylation status in IDC and ILC (P-value from Wilcoxon test). Right: PCA of these four proteins in 10 paired breast cancer tissues. Red, IDCs; green, ILCs. **(G)** Expression of the MAP1B, SC22B and CAVN1 proteins and their phosphorylation status in IDC and ILC (P-value from Wilcoxon test). **(H)** Expression of DREB, CALD1 and UTRO proteins and their phosphorylation status in IDC and ILC (P-value from Wilcoxon test). *, p<0.05; **, p<0.01.

To quantitatively investigate protein phosphorylation patterns in IDC and ILC tissues, the phosphoproteins derived from the IDC and ILC patient tissues were analyzed using the workflow shown in **Figure 3A**. A total of 560 phosphoproteins were all identified in greater than 60% of all paired luminal A breast cancer tissues with at least one phosphorylation site. These proteins were enriched in focal adhesion, MAPK and insulin signaling pathways (**Figure S7**). Similar to the proteomics results, differential phosphoproteins between IDC and ILC were screened by fold change (FC). In addition, 27 and 30 phosphoproteins were significantly differentially detected between IDC and ILC. Among these differential phosphoproteins, only two proteins (DCLK1 and FLNC) overlapped in both IDC and ILC, in which DCLK1, a kinase mostly involved in calcium-signaling, was significantly upregulated in IDC and downregulated in ILC, while FLNC, a large actin-cross-linking protein, was significantly downregulated in both IDC and ILC compared with their NATs (**Figures 3B, C**). These results indicated that differences in protein phosphorylation are more obvious than differences in protein expression. Among these significantly differential proteins/phosphoproteins, only ten differential phosphoproteins were also characterized as differential proteins by proteomics (**Figures 3D, E**). From the heatmap, ten proteins were clustered into three groups: (1) those that were upregulated in both ILCs and IDCs, while phosphorylation of these proteins was upregulated in IDCs but downregulated in ILCs. The PCA confirmed that these criteria clearly separates IDC and ILC (**Figure 3F**); (2) those that were upregulated in both ILCs and IDCs, while phosphorylation of these proteins was downregulated in IDCs but not in ILCs (**Figure 3G**); and (3) those that were maintained in IDCs and upregulated in ILCs, while phosphorylation of these proteins was significantly downregulated and maintained in ILCs (**Figure 3H**). In brief, our study established a comprehensive landscape of luminal A breast cancer for two subtypes at the proteome and phosphoproteome levels.

### Probing kinase activation differences between IDC and ILC

**Figure 4:**
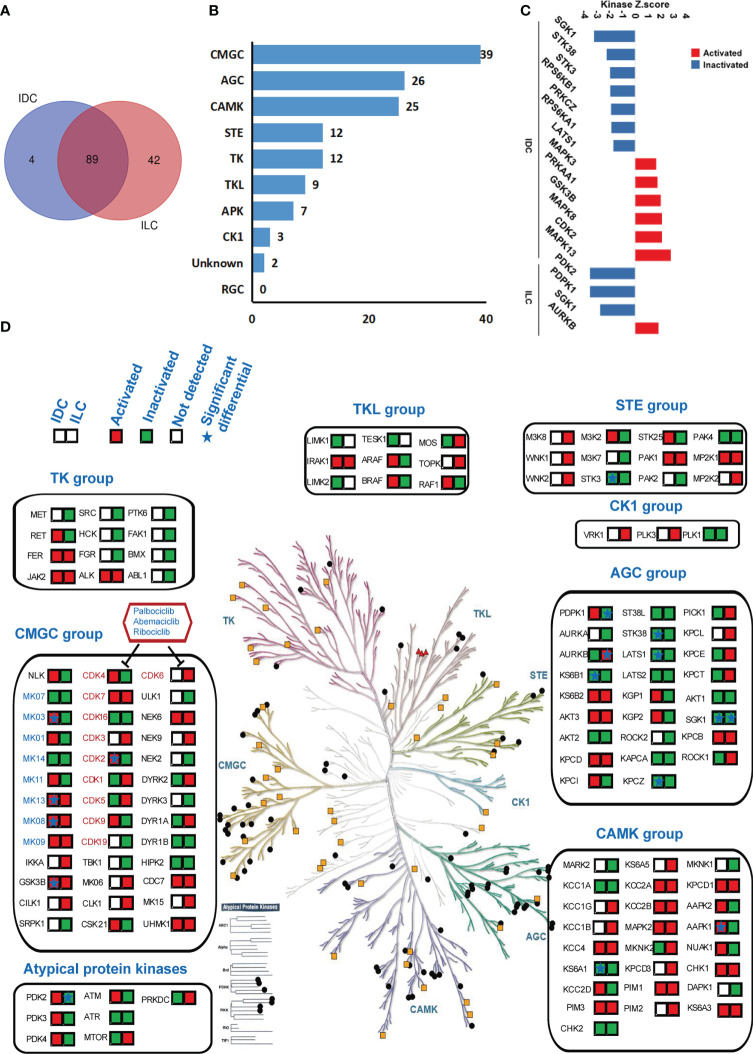
Kinase regulation in luminal A breast cancer by KSEA analysis. **(A)** Venn diagrams of identified kinases from IDCs and ILCs. Blue: IDCs; red: ILCs. **(B)** The distribution of 135 identified kinases in each group of human kinases. **(C)** Kinase regulation characterization in IDC and ILC. Kinases with a P-value < 0.05 based on the KSEA algorithm are shown. These kinases are predicted to be activated and inactivated in cancer tissue. **(D)** Compendium of the kinome detected in IDC and ILC under standard conditions. The colored panes with red or green showed the identified kinases in IDC and ILC. Red means the kinase is activated while green means the kinase is inactivated. The star in the panes means the difference of kinase is significant between IDC and ILC.

Site-selective phosphorylation of proteins is catalyzed by protein kinases (PKs), and these phosphorylation events play a fundamental role in regulating cellular functions. MS-based (phospho)proteomics provides a global and unbiased survey of kinase expression, activation and signaling. Many of the characterized protein functions in luminal A breast cancer participate in cancer-related signaling pathways, which are closely related to the phosphorylation and dephosphorylation of proteins and induce the activation or inhibition, respectively, of the signaling pathway. The phosphoproteomic shotgun experiment on luminal A breast cancer and NATs tissues detecting a total of >25,414 phosphorylation sites were examined to assess the activities of the kinases using Kinase-Substrate Enrichment Analysis (KSEA), and the KinMAP tool was used to analyze the location of kinases and to identify the location and distribution of the kinases in IDC and ILC (35, 41). The activities of 93 and 131 kinases were modulated in IDC and ILC, respectively. In addition, 89 of the kinases were commonly identified in both subtypes (**Figure 4A**). Kinases are categorized into ten groups based on statistical sequence analysis (42). The greatest number of identified phosphorylated protein kinases (39) belonged to the CMGC group, followed by the AGC group (26) and CAMK group (25) (**Figure 4B**). The activities of 13 and 4 kinases were predicted to be significantly different in IDC and ILC tissues compared to their activities in paired NATs (**Figure 4C** and **Figure S8**). In IDC, kinases MAPK13, MAPK3, MAPK8, CDK2, and GSK3B, which belong to the CMGC groups, were significantly activated, while SGK1, LATS1, KPCZ, STK38, and KS6B1, which belong to the AGC groups, were significantly inactivated. In addition, in ILCs, only one kinase, AURKB, which belongs to the AGC group, was significantly activated, while PDPK1 and SGK1 belong to the AGC group. PDK2, an atypical protein kinase, was significantly inactivated (**Figure 4D**). The target drugs for breast cancer were located in the CMGC group as inhibitors of CDK4/6, which are involved in the cell cycle control pathway and are checkpoints of G1/S phase. CDK2, which is also involved in the cell cycle control pathway, was significantly activated in IDC. Interestingly, the activities of PDPK1 and mTOR located in the mTOR signaling pathway were completely opposite between IDC and ILC (**Figure S9**).

## Discussion

The molecular characteristics of invasive ductal carcinoma (IDC) and invasive lobular carcinoma (ILC) in luminal A breast cancer remain elusive, and it remains challenging to distinguish them, especially late distant recurrence of ILC, which displays aggressive metastatic behavior associated with stage-matched IDC. Therefore, current clinical practice guidelines recommend similar treatment paradigms for both histologic subtypes (43), but the prognosis and survival of these two subtypes are different even they are both ER/PR positive and Her2 negative. Previous studies have revealed the influence of copy number variations (CNVs) on important cancer-related genes of IDC and ILC using genomics methods and found that over half of ILCs differed from IDCs in global transcription programs (17, 44). However, the distinct profiles at the proteome and phosphoproteome levels, which directly identify the difference between IDC and ILC, were not evaluated in previous studies. In this study, approximately 60% of proteins and phosphoproteins were commonly detected between the breast cancer and NATs, while approximately 65% of proteins and phosphoproteins were commonly between the IDC and ILC subtypes. Among the significantly differential proteins, greater than 99% exhibited the same tendency, and only 3 proteins displayed opposite behaviors between IDC and ILC compared with their NATs. Combining significantly differentially proteomes with phosphoproteomes, 10 proteins were selected and clustered into three groups, which contributed to distinguishing IDCs and ILCs with respect to protein and phosphorylation status (**Table 2**). From previous studies, we know that phosphoproteomics, especially cancer phosphoproteomics, provides comprehensive insights regarding kinases that are typically targeted for therapeutic applications (27). In our study, approximately 12,552 phosphopeptides were matched to 135 protein kinases with nine groups. This result indicates that markedly activated kinases were primarily located in the CMGC group, which is the most productive class of drug targets.

**Table 2:** Differential proteins and their phosphorylation between IDC and ILC compared with their respective NATs.

Protein Accession	Protein		Phosphorylation	
	IDC-FC	ILC-FC	IDC-FC	ILC-FC
H1X_HUMAN	1.78	0.56	ND	ND
CO4B_HUMAN	0.25	3.14	ND	ND
CRKL_HUMAN	0.63	3.26	ND	ND
CALD1_HUMAN	1.58	4.26	0.43	1.34
SC22B_HUMAN	3.99	4.11	1.80	2.44
CAVN1_HUMAN	0.63	0.74	0.18	0.23
ROA1_HUMAN	2.17	2.60	1.77	0.47
DREB_HUMAN	3.04	13.27	1.06	1.61
KTN1_HUMAN	3.78	4.90	3.36	0.41
SEPT2_HUMAN	1.83	2.43	3.82	0.67
UTRO_HUMAN	1.16	6.55	0.27	1.61
SEPT9_HUMAN	2.54	4.38	2.80	0.65
MAP1B_HUMAN	2.80	1.17	0.20	1.80

IDC, invasive ductal carcinoma; ILC, invasive lobular carcinoma; FC, Fold Change.

The most striking proteomics and phosphoproteomics features distinguished molecular features connecting the clinical, pathological, and prognostic features of ILC and IDC. In a previous study, the E-cadherin gene was identified as a marker to distinguish between IDC and ILC breast cancer, in which ILC may achieve invasive growth through loss of E-cadherin (15). In our proteomics results, we only observed E-cadherin in IDC tissue samples. From the different gene expression pattern studies, several genes, such as cathepsin B, survival, and TP11, were identified to distinguish between IDC and ILC. Expression of these genes like cathepsin B are elevated in many but not all cancers. However, the promiscuous expression of these genes especially for cathepsin B in tumor cells raises questions related to safety and specificity (45). Overall, it is difficult to distinguish between IDC and ILC at the gene expression level. In our study, protein expression levels of histone H1.10, complement C4-B, and Crk-like protein were significantly different between the IDC and ILC. Histone H1 is highly expressed, particularly in cancers, such as prostate cancer, and serves as a target for the delivery of the therapeutic drug doxorubicin (DOX) (46). It explained the differential sensitivity of DOX in the clinical treatment of IDC and ILC. Meanwhile, complement C4-B and Crk-like protein, which are involved in the immune system, were highly expressed in breast cancer, but differences between IDC and ILC are not well characterized. Crk-like protein can bind to the plexin-semaphoring-integrin (PSI) domain of β1 integrin and activate the mitogen-activated protein (MAP) kinase pathway, leading to nuclear transcription and enhanced cell division and migration (47). Septins are cytoskeletal proteins associated with GTP binding and participate in membrane interactions, which contribute to metastatic cancer cell dissemination and invasion and are mostly associated with the RAS oncogene. It has been reported that SEPT2 may impact two parallel pathways, p53/p21 and MEK/ERK, in cancer cells and mediate proliferation *via* regulation of cellular metabolic proteins (48, 49). Septins are related to the phosphorylation of MEK1/2 and downstream of ERK1/2, and loss of septin phosphorylation could lead to defects in morphogenesis and cytokinesis (50). Human CMGCs and AGC are known to regulate a variety of cell growth, proliferation, survival, and anti-apoptosis activities and extensively participate in the control of cell fate decisions; thus, they have been of interest in cancer research.

IDC and ILC tumors are currently treated similarly and have similar outcomes. The inability of unsupervised clustering to distinguish between the two tumor types suggests that many proteins were expressed in common, and subtle differences in protein expression may be responsible for the phenotypic differences that exist between these two subtypes. In particular, IDC and ILC exhibited striking differences in the expression of proteins associated with cell adhesion and invasion, suggesting that they may achieve invasive growth through distinct mechanisms, similar to the gene analysis. Our research provides insights to clarify the molecular characterization of IDC and ILC and contributes to discovering novel targets for further drug development and targeted treatment. However, because of strict filtering, the sample number is not huge enough. We will verify the candidate proteins carefully in the further study.

This study revealed that the proteome, phosphoproteome and kinome possess unique features and, when appropriately integrated, convey new insights and opportunities to discover novel biomarkers and even powerful drug targets.

## Data availability statement

The datasets presented in this study can be found in online repositories. The names of the repository/repositories and accession number(s) can be found below: MassIVE via accession ID: MSV000091530.

## Ethics statement

The studies involving human participants were reviewed and approved by Ethics Committee of Fujian Medical University Union Hospital (Fujian, China). The patients/participants provided their written informed consent to participate in this study.

## Author contributions

GY, X-DG, FF, and CYZ conceived and designed experiments. CYZ performed the majority of experiments. CYZ and XZ performed the majority of data and statistical analysis. GY, CYZ, and X-DG wrote and revised the manuscript. YL, HX, and MJ collected breast cancer tissues, clinical patient information and assisted data analysis. PW, Wenyan, and CRZ assisted sample processing of LC-MS. All authors contributed to the article and approved the submitted version.
