# Supplementary material for: Comprehensive proteome, phosphoproteome and kinome characterization of luminal A breast cancer
Source: Front Oncol. 2023 Mar 31;13:1127446. doi: 10.3389/fonc.2023.1127446 (PMC10102592; doi:10.3389/fonc.2023.1127446)
Supplement: Supplementary file 8 [file DataSheet_1.docx]

**Comprehensive proteome, phosphoproteome and kinome characterizaion of luminal A breast cancer**

Ganglong Yang^1,2#^, Chenyang Zuo^2,#^, Yuxiang Lin^3,4,5,#^, Xiaoman Zhou^2^, Piaopiao Wen^2^, Chairui Zhang^2^, Han Xiao^6^, Meichen Jiang^6^, Morihisa Fujita^2^ , Xiao-Dong Gao^1*^, and Fangmeng Fu^3,4,5*^

*^1^*The Key Laboratory of Carbohydrate Chemistry & Biotechnology, Ministry of Education; School of Biotechnology, Jiangnan University, 214122, Wuxi, China

*^2^*State Key Laboratory of Biochemical Engineering, Institute of Process Engineering, Chinese Academy of Sciences, 100190, Beijing, China

*^3^*Department of Breast Surgery, Fujian Medical University Union Hospital, 350001, Fuzhou, Fujian Province, China

*^4^*Department of General Surgery, Fujian Medical University Union Hospital, 350001, Fuzhou, Fujian Province, China

*^5^*Breast Cancer Institute, Fujian Medical University, 350001, Fuzhou, Fujian Province, China

*^6^*Department of Pathology, Fujian Medical University Union Hospital, 350001, Fuzhou, Fujian Province, China

# These authors contributed equally

*Corresponding author: Dr. Xiao-Dong Gao at xdgao@ipe.ac.cn

Dr. Fangmeng Fu at ffm@fjmu.edu.cn

**Supplementary figures**


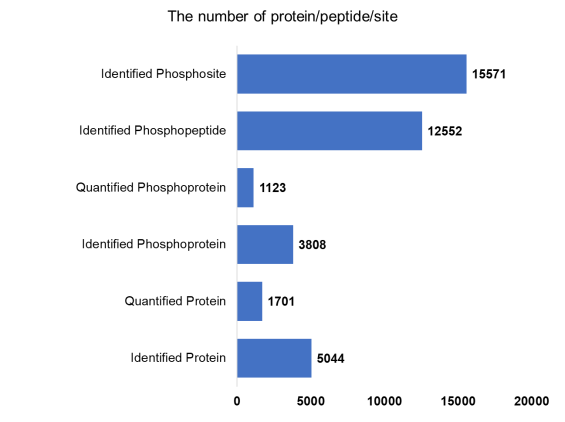

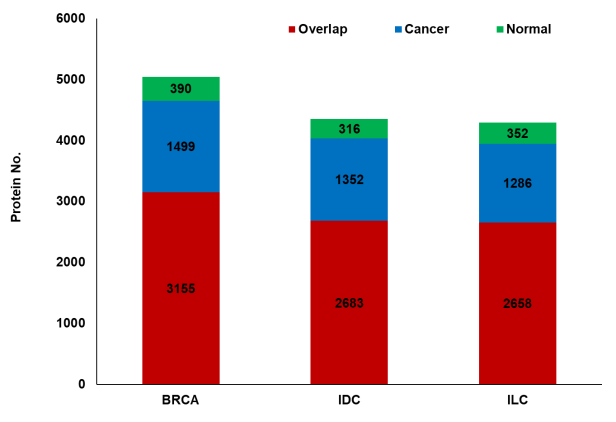

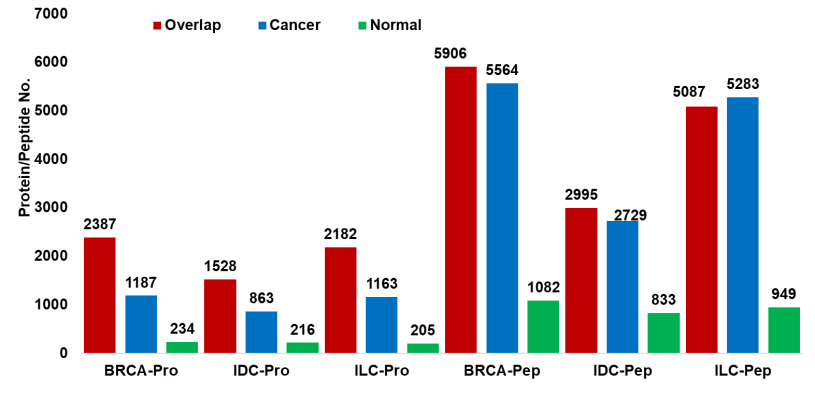

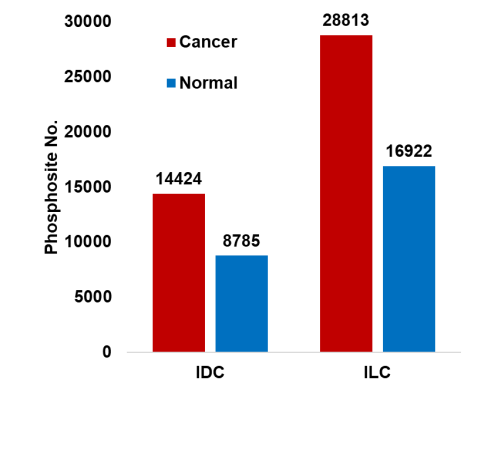

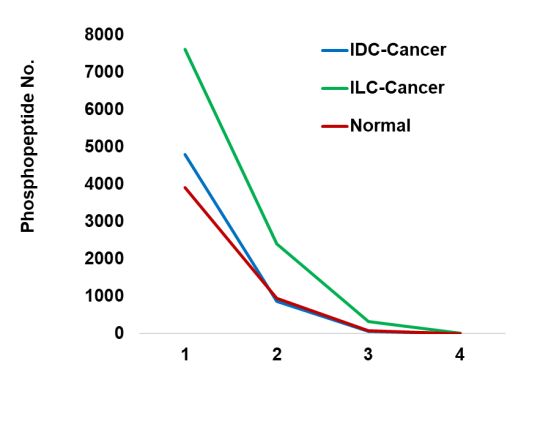


**a**

**b**

**c**

**d**

**e**

**Figure S1: Identification of tumor-associated proteins and their phosphorylation status comparing tumor and noncancerous adjacent tissues (NATs).** (a) Overview of identified proteins and phosphoproteins. (b) Identified proteins and their distributions in the whole breast cancer, IDC and ILC tissues. (c) Identified phosphoproteins, phosphopeptides and their distributions in the whole BRCA, IDC and ILC tissues. (d) Phosphosite distributions in IDC and ILC. (e) Phosphopeptides with different numbers of phosphosites between IDC and ILC. (f) Overview of protein/phosphoprotein distribution in breast cancer tissues


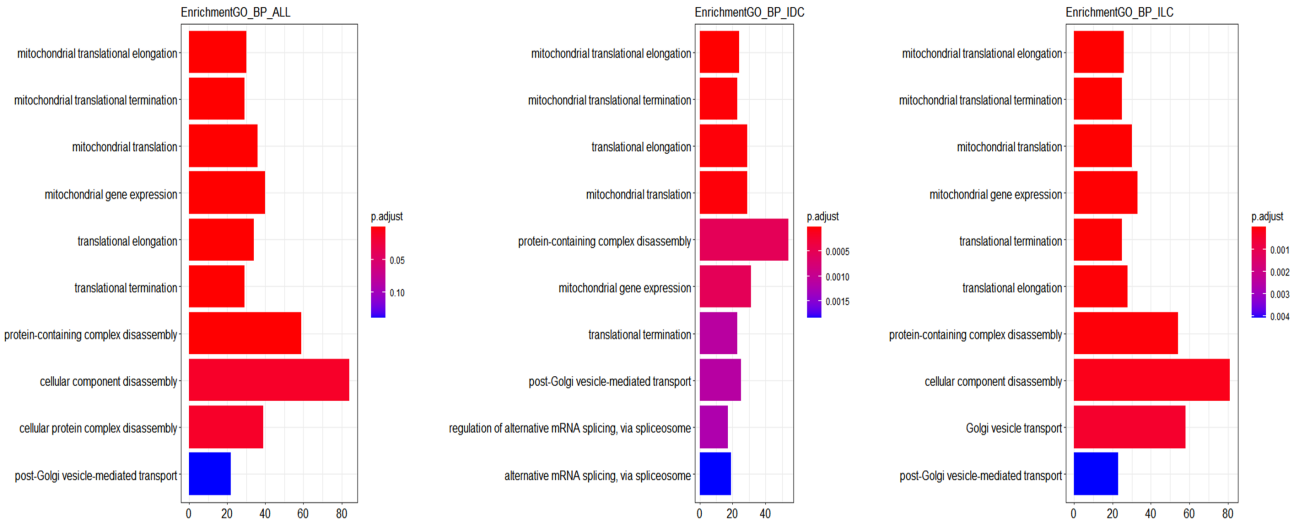


**a**

**b**

**c**

**Figure S2:**

1. **GO_BP result of the proteins identified in the whole breast cancer tissues**
2. **(C) GO_BP results of the proteins identified in the IDCs and ILCs subtypes**


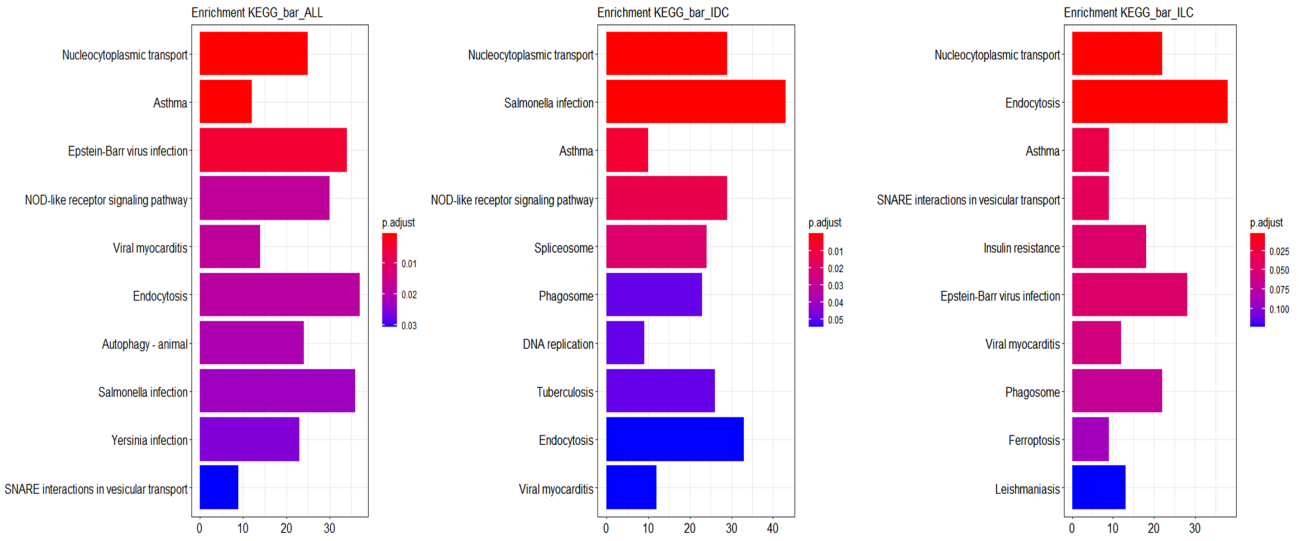


**a**

**b**

**c**

**Figure S3**:

1. **KEGG result of the proteins identified in the whole breast cancer tissues**
2. **(C) KEGG results of the proteins identified in the IDCs and ILCs subtypes**

**b**

**c**

**a**


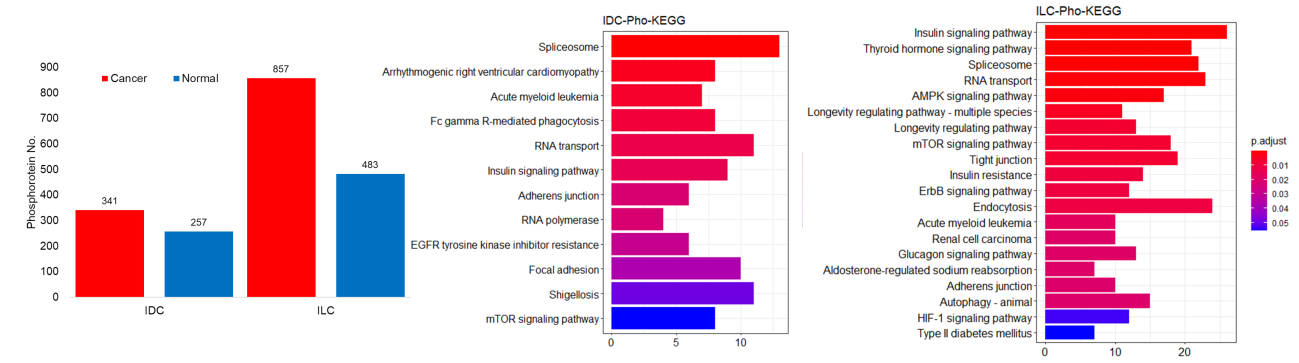


**Figure S4**:

1. **The number of phosphoproteins with ≥2 phosphosites simultaneously modified In IDCs or ILCs.**
2. **(C) KEGG result of the phosphoproteins identified with ≥2 phosphosites simultaneously modified in the IDCs or ILCs.**


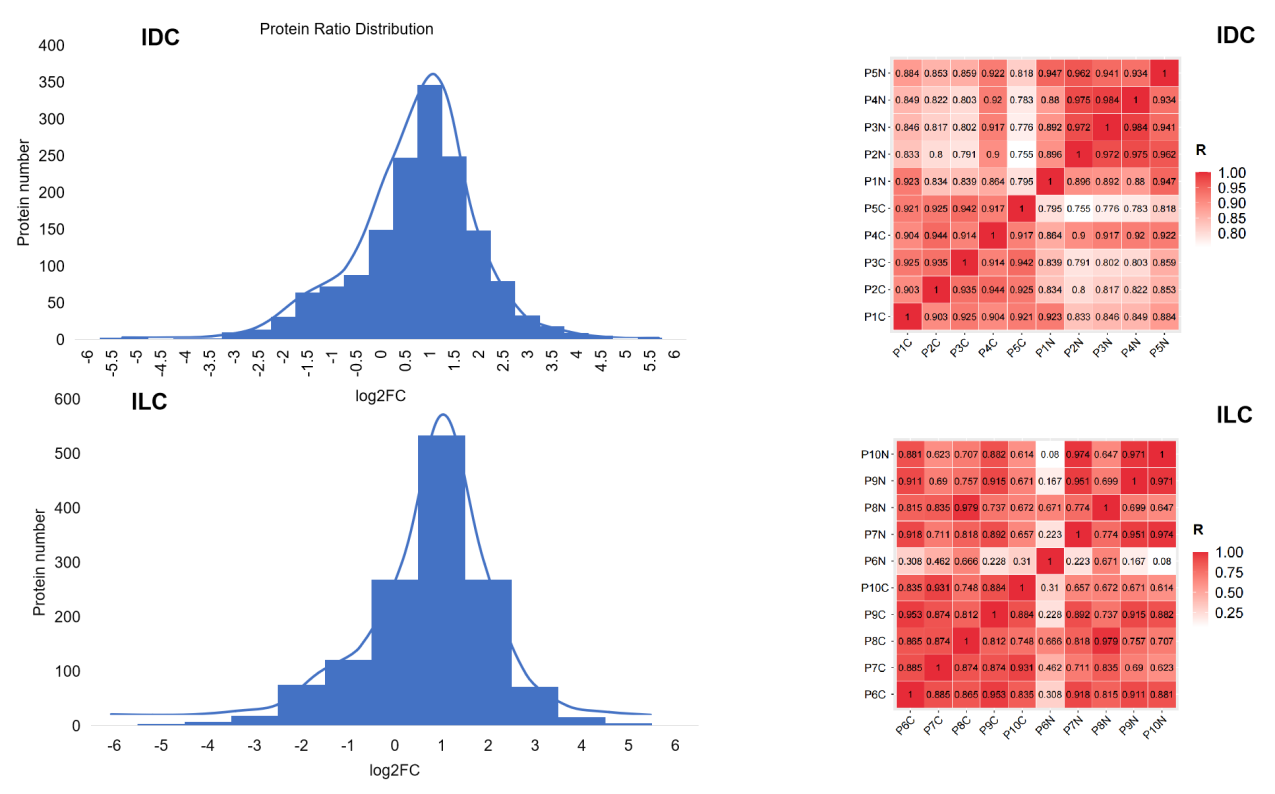


**a**

**b**

**Figure S5**:

1. **Normal distribution analysis of proteomics results**
2. **Correlation matrix of IDCs or ILCs proteomes (Spearman’s correlation coefficients)**


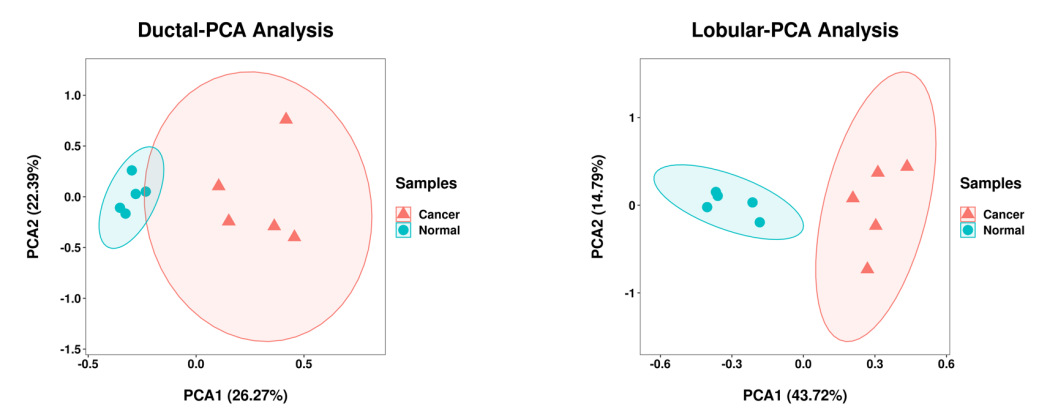


**Figure S6: The PCA analysis of cancer tissue and normal of IDC (left) and ILC (right).**


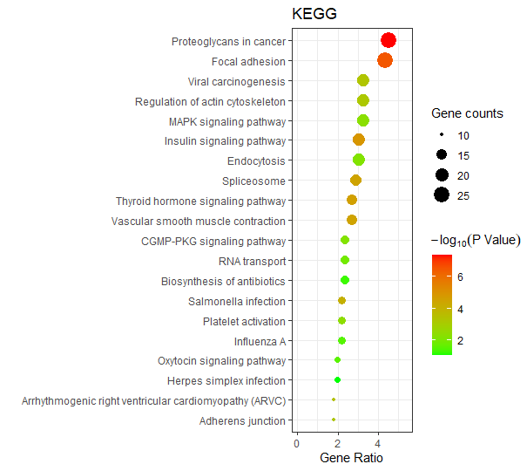


**Figure S7:** **KEGG analysis results of 560 phosphoproteins**


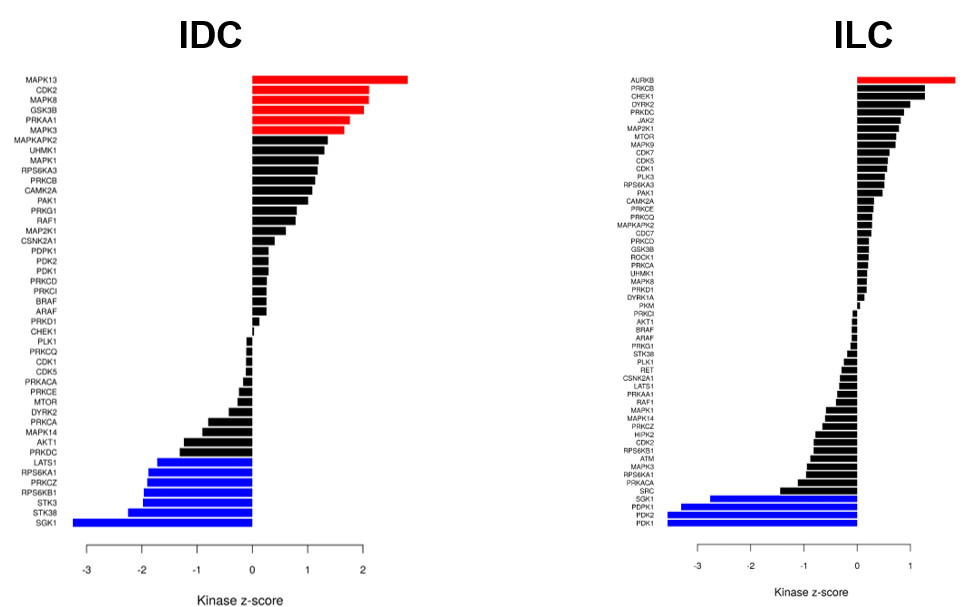


**Figure S8:** **Evaluation of kinase activities by KSEA in IDCs and ILCs across the phosphoproteomic results.**


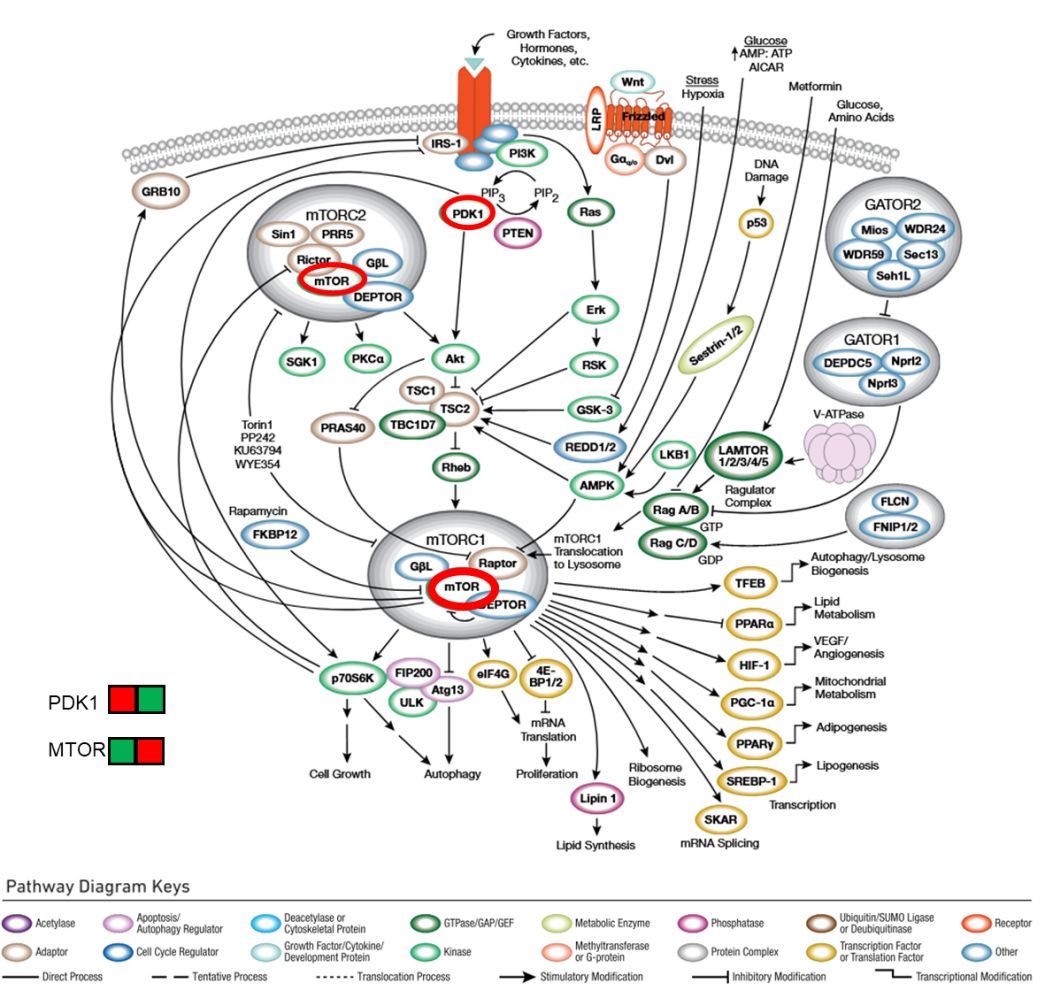


**b**


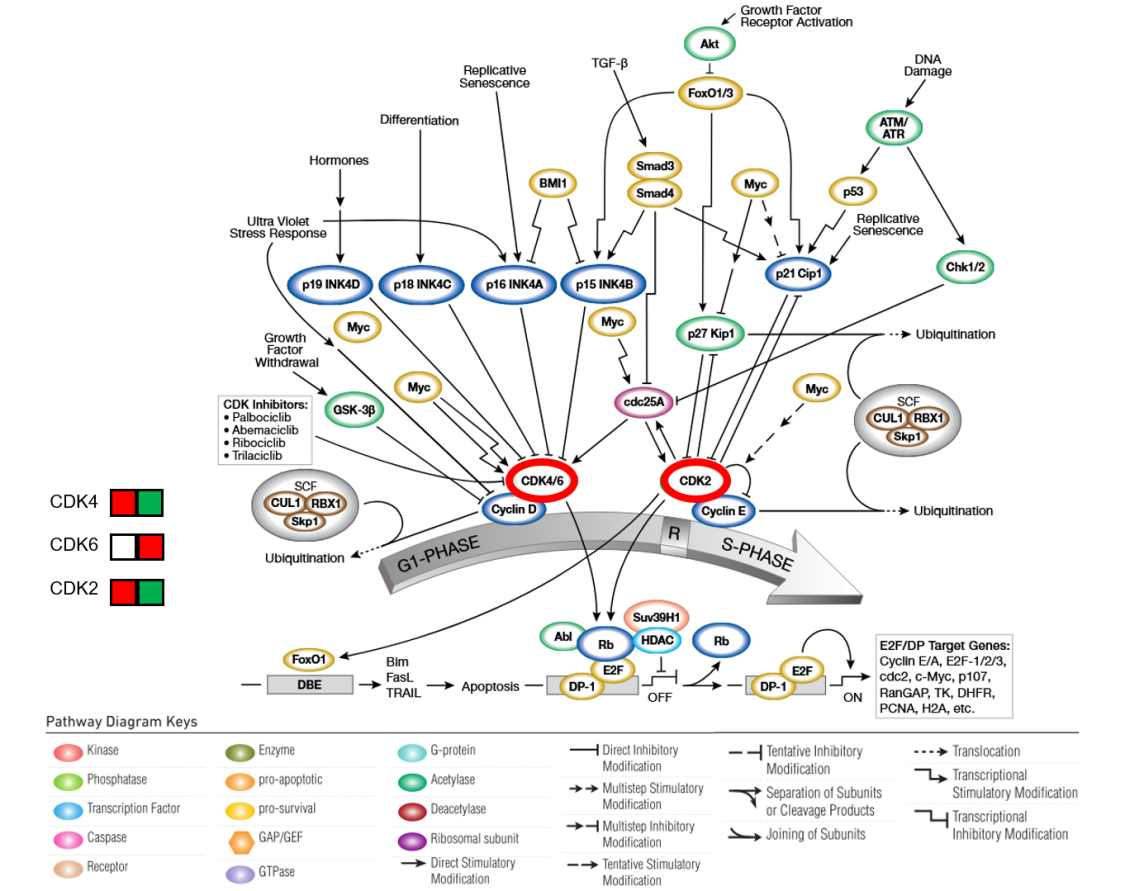


**a**

**Supplementary Figure S9: Selected important kinases detected in their activated (red) or inactivated (green) state in IDCs (right) or ILCs(left) are shown in the context of their molecular signaling pathway.**
